# Number of Polyploid Giant Cancer Cells and Expression of EZH2 Are Associated with VM Formation and Tumor Grade in Human Ovarian Tumor

**DOI:** 10.1155/2014/903542

**Published:** 2014-06-15

**Authors:** Li Zhang, Po Ding, Hongcheng Lv, Dan Zhang, Guang Liu, Zhengduo Yang, Yan Li, Jun Liu, Shiwu Zhang

**Affiliations:** ^1^Department of Pathology, Tianjin Union Medicine Center, Tianjin 300121, China; ^2^Department of General Surgery, Tianjin Union Medicine Center, Tianjin 300121, China; ^3^Department of Gynaecology and Obstetrics, Tianjin Union Medicine Center, Tianjin 300121, China; ^4^Department of Medical Imaging, Tianjin Union Medicine Center, Tianjin 300121, China

## Abstract

To investigate the associations among the number of polyploid giant cancer cells (PGCCs) and vasculogenic mimicry (VM), EZH2 expression, and serous ovarian tumor grade, a total of 80 paraffin-embedded serous ovarian tumor samples including 21 cases of primary carcinoma and their metastatic tumors, 26 cases of primary carcinoma without metastasis, and 12 cases of serous borderline cystadenoma were analyzed. PGCCs and VM were detected in human serous ovarian tumor. The metastatic foci of ovarian carcinoma had the highest number of PGCCs and VM. The number of PGCCs and VM increased with the grade of ovarian carcinomas. PGCCs generated erythrocytes via budding and together they formed VM. Tumor cells and cancer-associated fibroblasts were positive for EZH2 immunohistochemical staining. The tumor cells and cancer associated fibroblasts in the metastatic foci had the highest staining index of EZH2 staining. Both tumor cells and cancer-associated fibroblasts express EZH2 which then contributes to the malignant grade of serous ovarian tumor.

## 1. Introduction

Polyploid giant cancer cells (PGCCs) are a special subpopulation of cancer cells that contribute to solid tumor heterogeneity [1, 2]. PGCCs are the most commonly described histological features in the pathologic diagnosis of tumors. The shape of PGCC nuclei was irregular and the size of PGCC nuclei was at least three to five times larger than those in regular diploid cancer cells [3]. The formation and function of PGCCs are largely undefined and PGCCs were once considered an intermediate product of genomic instability [4, 5]. Our previous study confirmed that PGCC formation could be induced by hypoxia and the PGCCs then contributed to the generation of cancer stem-like cells [3]. PGCCs differ remarkably from diploid cancer cells in morphology, size, tumorigenic ability, radioresistance, and chemoresistance. PGCCs may contribute to tumor maintenance and recurrence because PGCCs can be considered seed cells. The number of PGCCs varies with the malignant grade of a tumor [3].

Enhancer of zeste homolog 2 (EZH2) is a histone-lysine N-methyltransferase [6] and plays a key role during embryogenesis [7]. Indeed, EZH2 can directly methylate the promoters of transcription factors that are essential for sustaining stem cell pluripotency [8]. Because EZH2 expression is important in many kinds of cancer stem cells, we detected EZH2 expression in PGCCs and examined its association with the malignant grade of human serous ovarian tumor.

It is reported that there are three kinds of tumor supply patterns including endothelium-dependent vessels (EVs), mosaic vessels, and vasculogenic mimicry (VM) [9]. VM channels are formed by tumor cells and a transitional blood supply pattern that satisfies the needs of a rapidly growing tumor and is eventually replaced by EVs. Sun et al. reported that hypoxia inducible factor-1*α* plays an important role in VM formation [10]. It has been more than ten years since Maniotis et al. reported VM for the first time and some detailed processes of VM formation remain unclear [11], including the source of erythrocytes in VM before VM connects with EVs. Bone marrow has generally been considered the source of these erythrocytes. We have previously reported that PGCCs of the BT-549 breast cancer cell line were able to generate erythrocytes expressing fetal hemoglobin both in vitro and in vivo [12]. Intact erythrocytes containing a complex mixture of embryonic, semiembryonic, and fetal hemoglobins have been shown to bind O_2_ strongly to satisfy the transitional need of cancer cells in a hypoxic microenvironment [12]. It has also been reported that human embryonic stem cells and induced pluripotent stem cells can also generate these cells in vitro [13–16]. Szabo et al. demonstrated the ability to generate multilineage blood progenitors from human dermal fibroblasts without establishing pluripotency [14]. Tumor cells can generate erythrocytes, which indicates that tumor cells and their newly generated erythrocytes can form VM during tumor development [17].

## 2. Materials and Methods

### 2.1. Tissue Samples

Paraffin-embedded human serous ovarian tumor tissue samples (*n* = 80) were randomly obtained from the Tumor Tissue Bank of Tianjin Union Medicine Center. These samples were collected from 2005 to 2013. None of the patients had been treated before surgical removal of the tumor. Tumor groups are given in the supporting information and the diagnosis was verified by two pathologists. The criteria of ovarian cancer grade system were according to the report of Malpica et al. who evaluate a two-tier system for grading ovarian serous carcinoma based primarily on the assessment of nuclear atypia and the mitotic rate [18]. We collected 26 cases of low grade ovarian cancer (group III primary ovarian tumor without metastasis) and 21 cases of high grade ovarian cancer (group II ovarian cancer with metastasis) in this study. The use of these tissues was approved by the institutional research committee, and the confidentiality of patient information has been maintained.

### 2.2. Tissue Microarray

Formalin-fixed, paraffin-embedded tissues from these ovarian tumor samples were analyzed and stained with standard H&E, and tumor tissues without necrosis were chosen to make a tissue microarray with 1.5 mm cores (2.0 mm between cores). Two typical spots for each sample were chosen based on the H&E staining.

### 2.3. Immunohistochemical (IHC) and Histochemical Double Staining

IHC staining was carried out using avidin-biotin peroxidase methods as described previously [10]. The detailed information is given in Supplementary Material (available online at http://dx.doi.org/10.1155/2014/903542).

### 2.4. PGCC Counting and Definition

Full H&E slides were used for the PGCCs counting. PGCCs were not always uniformly distributed throughout the tissue section, as hot spots of PGCC distribution were often observed. Five microscopic fields including a hot spot in each tissue section were counted with ×400 magnification and the average was calculated. The size of the PGCC nuclei was measured using a micrometer and H&E section. We used the description given by Zhang et al. that characterized a PGCC as a cancer cell with a nucleus at least three times larger than that of a diploid cancer cell [3].

### 2.5. Quantification of VM and EV

Slides were double stained and the structures of different blood supply patterns were observed microscopically with ×400 magnification. The average was calculated for each blood supply pattern. Like the PGCC count, five microscopic fields including a hot spot in each tissue section were counted. Using the standard introduced by Weidner [19], capillary vessels and microvessels in the tumor that were stained with CD31 were counted. A vessel containing a single positively stained endothelial cell is counted as one EV. The wall of VM channels is lined with tumor cells and erythroid cells can be found in the VM [9].

### 2.6. Counting and Statistical Methods

EZH2 expression levels were quantified according to the method described by Sun et al. [20], and the detailed information is given in supplementary information.

### 2.7. Statistical Analysis

Statistical software SPSS 13.0 was used to evaluate the data in this study and *P* < 0.05 was defined as statistically significant. Detailed statistical methods are given in supporting information.

## 3. Results

### 3.1. Number of PGCCs Associated with Histologic Characteristics of Human Serous Ovarian Tumor

**Figure 1:**
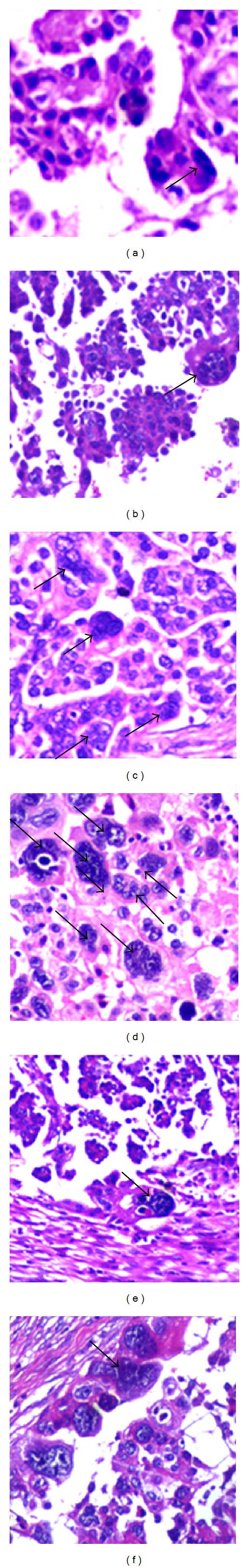
Identification of PGCCs in human serous ovarian tumor. (a) PGCCs in borderline serous cystadenoma (black arrow, H&E ×200). (b) PGCCs in human primary malignant serous ovarian carcinoma without metastasis (black arrow, H&E ×200). (c) PGCCs in malignant serous ovarian carcinoma with metastasis (black arrows, H&E ×200). (d) PGCCs in the metastatic tumor of serous ovarian carcinoma (black arrows, H&E ×200). (e) PGCCs located in the boundary of infiltration between normal and tumor tissue in ovarian carcinoma without metastasis (black arrow, H&E ×200). (f) PGCCs located in the boundary of infiltration between normal and tumor tissue in ovarian carcinoma with metastasis (black arrow, H&E ×200).

Using the characteristics of PGCCs set by Zhang et al. [3], results of micrometer measurements and morphologic observation indicated significant presence of PGCCs in human serous ovarian tumors with giant or multiple nuclei (Figure 1). The shape of PGCC nuclei was irregular, and the size of PGCC nuclei was three to five times larger than those in regular diploid cancer cells in borderline serous cystadenoma (Figure 1(a)). However, in ovarian carcinoma tissue and metastatic tumors, the size of the PGCC nuclei even reached 10–20 times that of the nuclei in regular diploid cancer cells (Figures 1(b), 1(c), and 1(d)). The majority of the PGCCs are seen around necrotic areas and in the boundary of infiltration between normal and tumor tissues. In the boundary, single PGCCs invade into the normal tissue (Figures 1(e) and 1(f)). Group II had the highest number of PGCCs and group IV had the lowest, and the differences among the groups are statistically significant (*χ* ^2^ = 49.55, *P* = 0.000) (Table 1). The average number of PGCCs is higher in group II than in group I (*Z* = −4.015, *P* = 0.000), higher in group I than in group III (*Z* = −2.600, *P* = 0.009), and higher in group III than in group IV (*Z* = −4.728, *P* = 0.000).

### 3.2. VM Present in Human Serous Ovarian Tumor

**Figure 2:**
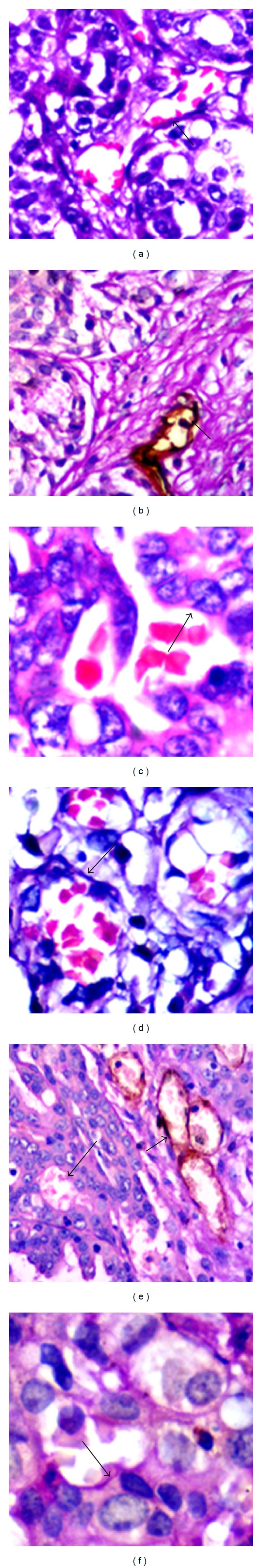
VM and EV present in human serous ovarian tumor. (a) EVs in human serous ovarian carcinoma (black arrow, H&E ×200). (b) Endothelial cells in EV were positive for CD31 and PAS staining (black arrows, double staining ×200). (c) VM without basement membrane (black arrow, H&E ×200). (d) VM with basement membrane in human serous ovarian carcinoma (black arrow, H&E ×200). (e) VM without basement membrane was negative for CD31 and PAS staining (large black arrow, double staining ×200) and EVs were positive for CD31 and PAS staining (small black arrow, double staining ×200). (f) VM with basement membrane was negative for CD31 staining and positive for PAS staining (black arrow, double staining ×200).

VM is an alternate tumor microcirculation pattern and is often present in certain high grade malignant tumors including inflammatory breast cancer [21], prostate cancer [22], and hepatocellular carcinoma [23]. To detect VM in human ovarian carcinoma and to determine if the number of VM channels is associated with histologic grade, VM structures were identified by H&E, IHC, and histochemical double staining. Results of H&E and double staining for CD31 and PAS showed that EVs with spindle endothelial cells were present in the serous ovarian carcinoma (Figure 2(a)). The spindle cells were positive for CD31 and the basement membrane of EVs was positive for PAS staining (Figure 2(b)). The walls of VM channels are made of tumor cells and red blood cells, and basement membrane may or may not be present. No necrosis or inflammatory cells were detected around VM structures. Some VM channels were negative for PAS staining (Figures 2(c) and 2(e)) and some VM channels were positive for PAS staining (Figures 2(d) and 2(f)).  Figure 2(e) shows CD31 positive EVs and CD31 negative VM structures coexisting in ovarian tumor tissue. For CD31 IHC staining, PBS was used as the first antibody for the negative control (Supplementary Figure 1(a)). To further verify the correlation between VM and the biological behavior of human serous ovarian tumor, we compared the number of VM structures in the four groups. Similar to the number of present PGCCs, group II had the highest number of VM channels and group IV had the lowest. Statistical analysis showed that the differences among these groups had statistical significance (*χ* ^2^ = 24.489, *P* = 0.000) (Table 2). The average number of VM is higher in group I than in group IV (*Z* = −4.225, *P* = 0.000), higher in group II than in group IV (*Z* = −4.337, *P* = 0.000), and higher in group III than in group IV (*Z* = −3.843, *P* = 0.000).

### 3.3. VM Structures Can Be Formed by PGCCs and Their Newly Generated Erythrocytes

**Figure 3:**
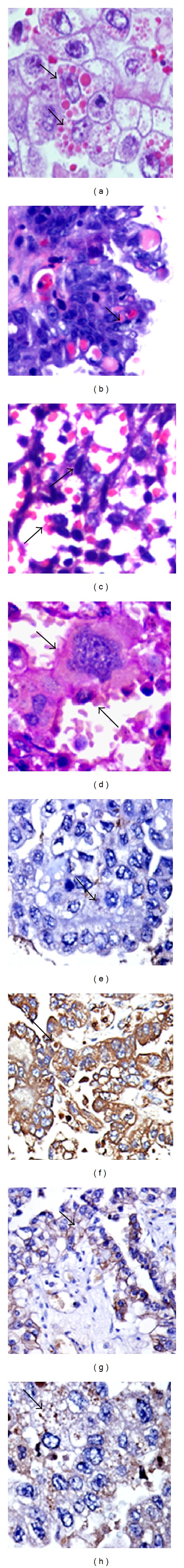
Human serous ovarian cancer cells generate erythrocytes. ((a) and (b)) Multiple red cell-like bodies around and within human ovarian cancer cells (black arrows, H&E ×200). (c) Many erythrocytes seen adhering to the surface of cancer cells (black arrows, H&E ×200). (d) Erythrocytes budding from PGCCs (black arrow, H&E ×200). (e) Red cell-like bodies in the cytoplasm of ovarian carcinoma cells were negative for hemoglobin-*α* (black arrow, IHC ×200). (f) Positive IHC staining for hemoglobin-*β*/*γ*/*ε*/*δ* in the red cell-like bodies (black arrow, IHC ×200). (g) Red cell-like bodies negative for fetal hemoglobin staining (black arrow, IHC ×200). (h) Red cell-like bodies were positive for hemoglobin-*ζ* IHC staining (black arrow, ×200).

We previously reported that the formation of PGCCs has properties of cancer stem cells [3]. PGCCs can generate erythrocytes both in vitro and in vivo [17]. H&E staining confirmed that there were many red cell-like bodies around and within human ovarian cancer cells. These bodies were located in the cytoplasm or adhered to the surface of cancer cells (Figures 3(a) and 3(b)).  Figure 3(c) shows that PGCCs and their newly generated erythrocytes can form VM structures and PGCCs line the lumen of VM channels.  Figure 3(d) shows a giant cancer cell with many erythrocytes budding from it. Staining with different hemoglobin antibodies was used to evaluate these red cell-like bodies. These bodies were positive for hemoglobin-*β*/*γ*/*δ*/*ε* (Figure 3(f)) and hemoglobin-*ζ* (Figure 3(h)) but negative for hemoglobin-*α* (Figure 3(e)) and fetal hemoglobin (Figure 3(g)). It should be emphasized that IHC staining of hemoglobin showed that tumor cells expressed hemoglobin-*β*/*γ*/*δ*/*ε* and fetal hemoglobin (Figures 3(f) and 3(g)). For different hemoglobins IHC staining, PBS was used as the first antibody for negative control (Supplementary Figure 1(B)–a to -d).

### 3.4. EZH2 Expression in Cancer Cells and Fibroblasts in Human Ovarian Tumors

**Figure 4:**
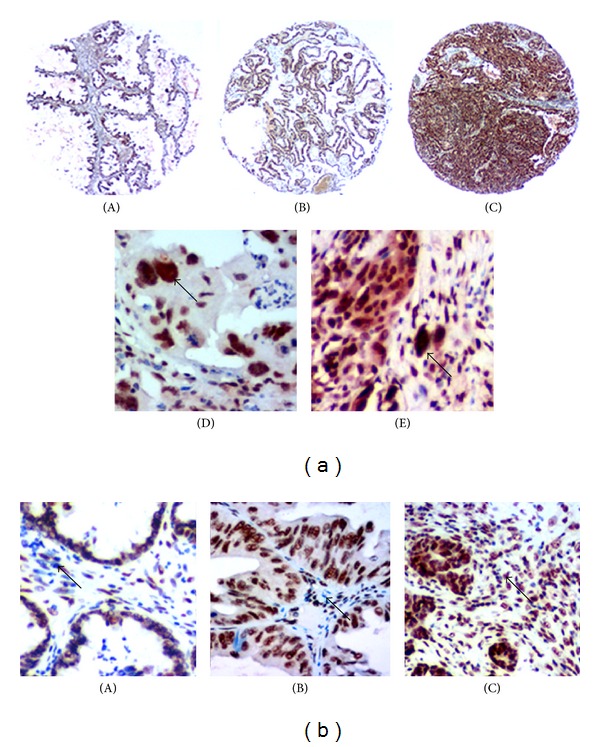
EZH2 expression in human serous ovarian tumor cells and CAF. (a) EZH2 expression in human serous ovarian tumor cells. (A) EZH2 expression in borderline serous cystadenoma (IHC ×100). (B) EZH2 expression in human serous ovarian cancer cells without metastasis (IHC ×100). (C) EZH2 expression in human serous ovarian cancer cells with metastasis (IHC ×100). (D) EZH2 expression in the metastasis of human serous ovarian carcinoma; PGCCs are positive for EZH2 staining (black arrow, IHC ×200). (E) EZH2 expression in the PGCCs located in the boundary between tumor tissue and normal tissue (black arrow, IHC ×200). (b) EZH2 expression in the CAF of human serous ovarian tumor. (A) In group IV (black arrow, IHC ×200). (B) In group III (IHC ×200). (C) In group I (IHC ×200).

Our previous studies have shown that EZH2 protein is overexpressed in PGCCs using iTRAQ-based proteomic analysis comparing PGCCs and diploid cancer cells [24]. To verify the expression of EZH2 and its association with tumor biological behaviors, IHC staining for EZH2 was performed. The 80 cases of serous ovarian tumor were made into a tissue microarray. PBS was used as the first antibody for negative control of EZH2 IHC staining (Supplementary Figure 1(c)). Positive staining for EZH2 was localized in the nuclei of tumor cells and cancer-associated fibroblasts (CAF).  Figure 4(a) shows the results of IHC staining for EZH2 protein. The metastatic cancer cells in group II had the highest staining index for EZH2, and the borderline serous cystadenoma had the lowest (*χ* ^2^ = 11.276, *P* = 0.010) (Table 3). Statistical analysis showed that the expression of EZH2 in metastatic cancer cells (Figure 4(D)) was higher than that in the ovarian carcinoma without metastasis (Figure 4(B)) (*Z* = −3.154, *P* = 0.002) and cystadenoma (Figure 4(A)) (*Z* = −2.704, *P* = 0.007) (Figure 4(C)). The PGCCs, especially single PGCCs located in the border of infiltration, were also positive for EZH2 IHC staining (Figure 4(E)).

EZH2 protein is also expressed in the CAF and shows a tendency to increase expression as malignancy increases in serous ovarian tumors. Figure 4 presents the EZH2 expression in cystadenoma and ovarian carcinoma with and without metastasis. CAF in metastatic ovarian carcinoma had the highest EZH2 expression of the groups assessed (*χ* ^2^ = 26.945, *P* = 0.000) (Table 4). There were statistically significant differences between group I and group II (*Z* = −3.203, *P* = 0.001), group II and group III (*Z* = −4.814, *P* = 0.000), and group II and group IV (*Z* = −3.785, *P* = 0.000).

## 4. Discussion

The majority of ovarian carcinomas are of the epithelial type [25], and serous ovarian carcinomas comprise more than half the diagnosed cases of ovarian carcinoma [25]. We present evidence for the first time that PGCCs and VM exist in human serous ovarian tumors, the number of PGCCs and VM structures is associated with malignancy, and PGCCs generate erythrocytes that can help form VM channels. Immunohistochemical detection showed that tumor cells and CAF expressed EZH2 in the nucleus, similar to previous reports in glioblastoma, non-Hodgkin lymphoma, and nasopharyngeal carcinoma [26, 27].

The nuclear features of a tumor cell are some of the most commonly described histopathology features of human tumors, and all these features typically become more prominent as the pathologic grade and disease stage increase [18, 28, 29]. Nuclear atypia has been used to make prognoses for numerous tumor types, including ovarian carcinoma [18, 30]. In this study, more PGCCs were detected in high grade malignant tumor than in low grade malignant tumor. Most of the PGCCs were located around necrotic areas and in the boundary between normal and tumor tissues where tumor cells are in a hypoxic microenvironment. As in physiological hypoxia, our previous study has confirmed that hypoxia chemically mimicking cobalt chloride also induces the formation of PGCCs in vitro [3]. PGCCs have properties found in cancer stem cells and may present the cellular basis for the generation of stem cells. These same features may also contribute to drug resistance. Many kinds of stresses including radiotherapy and chemotherapy (cisplatin, paclitaxel, etc.) can induce the formation of PGCCs [31, 32].

Hypoxia can increase self-renewal of cancer stem cells and promote stem cell-like phenotype expression [33–35]. Aside from inducing the formation of PGCCs, hypoxia also plays an important role in the formation of VM. The normal response to hypoxia is to stimulate the growth of new blood vessels. Hypoxia can activate some invasion- and metastasis-associated tumor genes including erythropoietin [36], vascular endothelial growth factor (VEGF), and the VEGF receptor Fit-1 [37], enabling the cells to become more invasive and form VM structures in poor conditions. Similar to the pattern seen with PGCCs, the number of VM structures is also highest in the metastases of ovarian carcinomas. These VM channels can connect with EVs to achieve adequate blood supply. Results of our study confirm that many red cell-like bodies are located in the cytoplasm or around the PGCCs and cancer cells in the VM structures and these red cell-like bodies express hemoglobin-*β*/*γ*/*ε*/*δ* and hemoglobin-*ζ* as detected by IHC staining. Hemoglobin-*ζ* is *α*-like hemoglobin and hemoglobin-*ζ* polypeptide is synthesized in the yolk sac of the early embryo, while hemoglobin-*α* is produced throughout fetal and adult life [38]. He and Russell reported that coexpression of hemoglobin-*ζ*_2_ *β*_2_^*S*^ can lead to a substantial improvement in the tissue oxygenation of mice [39] and the *ζ* hemoglobin chain has a higher oxygen affinity that may satisfy the transitional need of cancer cells in a hypoxic microenvironment [40]. Thus, VM structures can be formed by PGCCs or cancer cells and their newly generated erythrocytes with high O_2_ binding affinity.

Recent studies have confirmed the importance of EZH2 in maintaining the pluripotency of embryonic stem cells and activation of normal stem cells [41–43]. EZH2 may play a similar role in cancer stem cells [44, 45]. Furthermore, EZH2 is upregulated in a broad range of solid human malignancies, where its overexpression is associated with poor prognosis [46]. This study showed that serous ovarian tumor cells express EZH2 and increasing EZH2 expression is associated with increasing malignant grade of serous ovarian tumor. Single PGCCs invading normal tissue had strong EZH2 expression in the nucleus. Trenkmann et al. reported that EZH2 was downregulated in senescent fibroblasts [47] and overexpressed in rheumatoid arthritis synovial fibroblasts [48]. CAFs play a vital role in tumor initiation and progress [48] and EZH2 expression in CAFs may also be essential though the detailed molecular mechanisms need to be clarified.

## 5. Conclusion

Among gynecological malignancies, ovarian carcinoma is the leading cause of death and the second most common type overall [49]. There are more than two hundred thousand new cases of ovarian carcinoma diagnosed worldwide every year [50]. Our study provides a novel concept that VM and PGCCs are present in human serous ovarian tumors, the numbers of PGCCs and VM structures are associated with malignant grade, and PGCCs with their newly generated erythrocytes contribute to the formation of VM.

## Supplementary Material

The supplementary information provided the detailed information of tumor group, the staining method of the IHC and histochemical double-staining, the method of evaluating the results of IHC staining. Furthermore, figures of the negative control which PBS was used as the first antibody were also shown in the supplementary information.

## Figures and Tables

**Table 1:** Comparison of the average number of PGCCs in human ovarian tumors.

	Group	*n*	Number of PGCCs	*χ* ^2^	*P* value
Primary ovarian tumor	I	21	35.75 ± 23.54	49.55	0.000
Corresponding metastatic tumor	II	21	57.21 ± 24.88		
Primary ovarian tumor without metastasis	III	26	18.12 ± 8.70		
Borderline serous cystadenoma	IV	12	5.80 ± 1.89		

**Table 2:** Comparison of the average number of VM in human ovarian tumors.

	Group	*n*	Number of VM	*χ* ^2^	*P* value
Primary ovarian tumor	I	21	11.00 ± 8.41	24.489	0.000
Corresponding metastatic tumor	II	21	16.66 ± 16.69		
Primary ovarian tumor without metastasis	III	26	9.50 ± 8.93		
Borderline serous cystadenoma	IV	12	1.10 ± 2.15		

**Table 3:** Comparison of the EZH2 expression levels in human ovarian tumor cells.

	Group	*n*	Staining index of EZH2	*χ* ^2^	*P* value
Primary ovarian tumor	I	21	10.05 ± 2.42	11.276	0.010
Corresponding metastatic tumor	II	21	11.43 ± 1.43		
Primary ovarian tumor without metastasis	III	26	9.00 ± 2.95		
Borderline serous cystadenoma	IV	12	8.75 ± 3.65		

**Table 4:** Comparison of the EZH2 expression levels in cancer-associated fibroblasts.

	Group	*n*	Staining index of EZH2	*χ* ^2^	*P* value
Primary ovarian tumor	I	21	3.14 ± 2.10	26.945	0.000
Corresponding metastatic tumor	II	21	5.86 ± 2.37		
Primary ovarian tumor without metastasis	III	26	2.81 ± 1.23		
Borderline serous cystadenoma	IV	12	2.75 ± 1.60		

## Abbreviations

PGCCs: Polyploid giant cancer cells
EZH2: Enhancer of zeste homolog 2
PcG: Polycomb group
IHC staining: Immunohistochemical staining
VM: Vasculogenic mimicry
EV: Endothelium-dependent vessel
CD31: The platelet-endothelial cell adhesive molecule
PAS: Periodic acid-Schiff
CAF: Cancer-associated fibroblast
VEGF: Vascular endothelial growth factor.
