# Supplementary material for: Number of Polyploid Giant Cancer Cells and Expression of EZH2 Are Associated with VM Formation and Tumor Grade in Human Ovarian Tumor
Source: Biomed Res Int. 2014 Jun 15;2014:903542. doi: 10.1155/2014/903542 (PMC4082869; doi:10.1155/2014/903542)
Supplement: Supplementary file 1 — The supplementary information provided the detailed information of tumor group, the staining method of the IHC and histochemical double-staining, the method of evaluating the results of IHC staining. Furthermore, figures of the negative control which PBS was used as the first antibody were also shown in the supplementary information. [file 903542.f1.pdf]

Supporting Information：

Tumor group. The tumors included 21 cases of primary cancer (group Ⅰ) and their corresponding omental metastatic cancer (group Ⅱ), 26 cases of primary cancer without metastasis (group Ⅲ) and 12 cases of borderline serous cystadenoma which were low malignant potential and described as non-invasive tumors (group Ⅳ).

Immunohistochemical (IHC) and histochemical double-staining. Four-micrometer-thick sections were deparaffinized in xylene and hydrated in ethanol, and then antigen retrieved in10 mM sodium citrate buffer (pH 6.0) and endogenous peroxidase activity was blocked. After blocking nonspecific binding sites, sections were incubated overnight at 4°C with rabbit polyclonal anti-EZH2 (Zhongshan Inc., China, 1:100 dilution), monoclonal mouse anti-CD31 (MAB-0031, Maixin.Bio, China, 1:100 dilution), rabbit monoclonal anti-hemoglobin (Epitomics, USA, 1:100 dilution), goat polyclonal anti-hemoglobinβ/γ/δ/ε (Santa Cruz Biotechnology, USA, 1:100 dilution), goat polyclonal anti-fetal hemoglobin (Novus Biologicals, USA, 1:200 dilution) and rabbit polyclonal anti-hemoglobin-ζ (Fitzgerald, USA, 1:250 dilution) antibodies. Following incubation, the sections were incubated with biotinylated IgG for 20 min at 37°C followed by incubation with 3,3-diaminobenzidine chromogen for 1-3 min and then washed with distilled water. After IHC staining for CD31, the sections were then washed with running water for 5 min and incubated with periodic acid Schiff (PAS) reagent for 15 min. Finally, all of the sections were counterstained with hematoxylin. Normal human stomach mucous membrane was the positive control for PAS staining.

Counting and statistical methods. Both the intensity and the percentage of positive cells were evaluated. Tumor cells with brown nuclei were considered positive. For stain intensity, 1 was denoted for no stain, 2 was for weak positive with faint yellow, and 3 was for strong positive with brown staining. The number of positive cells was visually evaluated and cell expression was stratified as follows: 1 (negative) for <10% positive cells, 2 (weak) for 11-30% positive cells, 3 (moderate) for 31-50% positive cells, and 4 (strong) for >50% positive cells. The sum (staining index) of stain intensity and positive cell scores were used to determine the final result for each

section.

Statistical analysis. The Kruskal-Wallis test was used to compare the differences in PGCC number, VM number and EZH2 expression. Wilcoxon signed-rank test was performed to analyze the differences in PGCC number, VM number, and EZH2 expression. The Mann-Whitney Test was done to compare the differences in PGCC number, VM number and EZH2 expression.

Supplementary figure-1.

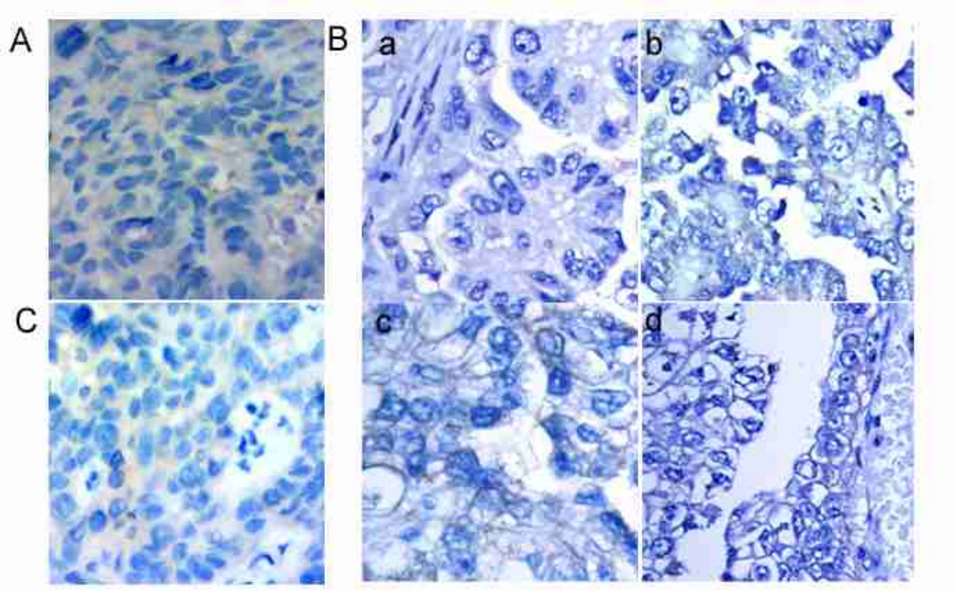

Supplementary figure 1. The negative control of IHC staining. A. PBS was used as the first antibody for negative control of CD31 IHC staining. B. Negative control of different hemoglobins IHC staining. a) Negative control of hemoglobin- IHC staining. b) Negative control of hemoglobin-β/γ/ε/δ IHC staining. c) Negative control of fetal hemoglobin IHC staining. d) Negative control of hemoglobin- IHC staining. C. PBS was used as the first antibody for negative control of EZH2 IHC staining.
